# Impact of Pulse Parameters of a DC Power Generator on the Microstructural and Mechanical Properties of Sputtered AlN Film with In-Situ OES Data Analysis

**DOI:** 10.3390/ma16083015

**Published:** 2023-04-11

**Authors:** Wei-Yu Zhou, Hsuan-Fan Chen, Xue-Li Tseng, Hsiao-Han Lo, Peter J. Wang, Ming-Yu Jiang, Yiin-Kuen Fuh, Tomi T. Li

**Affiliations:** 1 Department of Mechanical Engineering, National Central University, Taoyuan 320317, Taiwan; 2 Delta Electronics Incorporated, Taipei 100116, Taiwan

**Keywords:** Reactive pulsed DC magnetron sputtering, AlN, BoxBehnken method, Pulse parameter, In-situ OES, PCA, RSM, CatBoost modelling

## Abstract

In the present study, the sputtered aluminum nitride (AlN) films were processed in a reactive pulsed DC magnetron system. We applied a total of 15 different design of experiments (DOEs) on DC pulsed parameters (reverse voltage, pulse frequency, and duty cycle) with Box–Behnken experimental method and response surface method (RSM) to establish a mathematical model by experimental data for interpreting the relationship between independent and response variables. For the characterization of AlN films on the crystal quality, microstructure, thickness, and surface roughness, X-ray diffraction (XRD), atomic force microscopy (AFM), and field emission-scanning electron microscopy (FE-SEM) were utilized. AlN films have different microstructures and surface roughness under different pulse parameters. In addition, in-situ optical emission spectroscopy (OES) was employed to monitor the plasma in real-time, and its data were analyzed by principal component analysis (PCA) for dimensionality reduction and data preprocessing. Through the CatBoost modeling and analysis, we predicted results from XRD in full width at half maximum (FWHM) and SEM in grain size. This investigation identified the optimal pulse parameters for producing high-quality AlN films as a reverse voltage of 50 V, a pulse frequency of 250 kHz, and a duty cycle of 80.6061%. Additionally, a predictive CatBoost model for obtaining film FWHM and grain size was successfully trained.

## 1. Introduction

AlN is a versatile material with high sound acoustic wave velocity, high thermal conductivity, low thermal-expansion coefficient, wide band gap, and low dielectric constant which are significant technical benefits for the semiconductor industry. Reactive sputtering offers a promising method in film deposition at low temperatures with a deposited film of homogenous composition, superior substrate adhesion, and lower contamination [1,2,3,4]. Due to the excellent piezoelectric performance of highly C-axis oriented AlN films, they are widely used in the above-mentioned applications, making the production of highly c-axis oriented AlN films with low surface roughness a major goal [5]. In their study, the crystal quality of the films was evaluated using the FWHM of the AlN (002) peak, and pulse parameters corresponding to the lowest FWHM value were determined through experiments. A study investigated the effect of process parameters on residual stress in AlN films, which is crucial for their mechanical stability. Residual stress correlates with film thickness and crystallite orientation. In-situ OES data was analyzed using PCA to predict the stress state, and RSM was applied to determine the optimum conditions for minimizing residual stress [6]. Another study focused on developing a cost-effective process to produce solar coatings for solar receiver tubes in linear focusing concentrated solar power (CSP) technology. The reactive magnetron sputtering technology in transition mode was applied to deposit all ceramic constituents of solar coatings, which significantly improved deposition rates. The developed process allowed for a 44% reduction in energy consumption, and the produced coating exhibited excellent thermal stability [7]. Their work optimized process parameters for high Young’s modulus [002] oriented aluminum nitride (AlN) thin films by pulsed DC reactive sputtering, achieving Young’s modulus up to 335 GPa and a surface roughness of 1.2 nm. The optimal pulse parameters were a frequency of 100 kHz and a duty cycle of 80% [1]. Our previous study has investigated the effect of N2 gas flow and power on the minimum film residual stress of AlN thin films deposited by pulsed DC reactive sputtering on Si (100). Large-scale in-situ OES, XRD, SEM, TEM, FTIR, and alpha-step profiler data were analyzed to determine the film stress states. They proposed a residual stress (VRS) value calculated by a standard deviation in the first principal component direction (PC1-DEV) method. The study suggests a methodology using PCA to determine residual stress characterization as an in-situ plasma monitoring tool for the AlN thin film deposition process [2].

Plasma properties are studied to better understand plasma vacuum processing and improve process control and repeatability. Typically, duty cycles between 50 and 90 percent and pulse frequencies between 10 and 350 kHz are used throughout the deposition process [8]. Therefore, understanding the basics of the creation and behavior of pulsed DC magnetron discharges is required to master the new benefits of pulsed DC sputtering. Furthermore, to establish the connections between film properties from film measurements to pulse parameters, it is essential to explain relationships between them and film properties such as film structure, composition, and characteristics, which are particularly interesting for practical applications.

**Figure 1:**
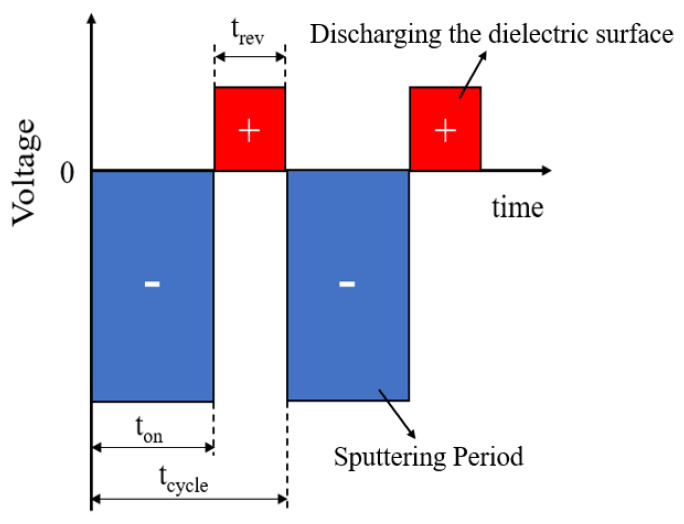
A typical pulsed DC sputtering pattern in an asymmetric bipolar voltage waveform.

Pulsed DC magnetron sputtering is a widely-used technology in the industry today, and it is especially effective for fabricating materials with excellent electrical insulation properties. It can obtain thin films with high density and good crystallinity at low temperatures [9,10,11,12]. In the current experiment, AlN films were processed by a reactive DC magnetron sputtering with an asymmetric bipolar-pulsed DC power source at various pulse settings in reverse voltage, pulse frequency, and duty cycle. The asymmetrical bipolar-pulsed DC power supply can adjust its pulse parameters according to requirements to improve efficiency. Compared with radiofrequency (RF) power supply, it can have a better deposition rate and prevents an electrically insulating layer from being deposited on the cathode surface by adding a reverse voltage, which can reduce charge buildup and arcing in affecting film quality [13]. A typical waveform and pulse feature was shown in Figure 1. This study used XRD, AFM, and FE-SEM to determine the microstructure of films. RSM, in conjunction with a mathematical Box–Behnken method [6,14], was employed to setup a DOE model for how the power supply DC pulse factors impact the microstructural properties of films.

In addition, this study trained thin film quality prediction models through PCA dimensionality reduction combined with the CatBoost model. The CatBoost classifier algorithm was adopted for training transmission line faults data in the multi-dataset from an electrical power system owing to its training speed, accuracy, and ability. The trained model implements an excellent accuracy of 99.54% [15]. The OES data have been widely built-in virtual metrology (VM) system. Multi-features in OES data are reduced dimensionality using principal component analysis (PCA) [16]. There is research that presents a non-invasive method for ensuring plasma parameters inside a radio-frequency ion thruster (RIT) by OES data incorporating PCA [17]. Data preprocessing with a machine learning or deep learning model is already common in artificial intelligence architecture. The PCA preprocessed the OES data, and the results were handed over to the multilayer perceptron (MLP) model for training [18]. The initialization of the variational autoencoder (VAE) used in MLP had the best result to predict plasma density of etch tool by OES data [19]. A real-time machine VM based on artificial intelligence has been developed. It can use OES data to supervise plasma nitridation processes in predicting the accuracy of electron temperature at 90% and electron density at 97% [20]. Another study presented generative adversarial networks (GANs) of deep learning to solve fault detection with imbalanced data in the plasma etching process. It verified the benefits of GANs solving imbalance OES data problems [21]. This study aims to identify the optimal pulse parameters for producing high-quality AlN films. Additionally, a predictive CatBoost model from PCA was successfully trained by collecting OES data for obtaining film FWHM and grain size.

## 2. Methodology

### 2.1. Experimental Setup and PVD Process

**Figure 2:**
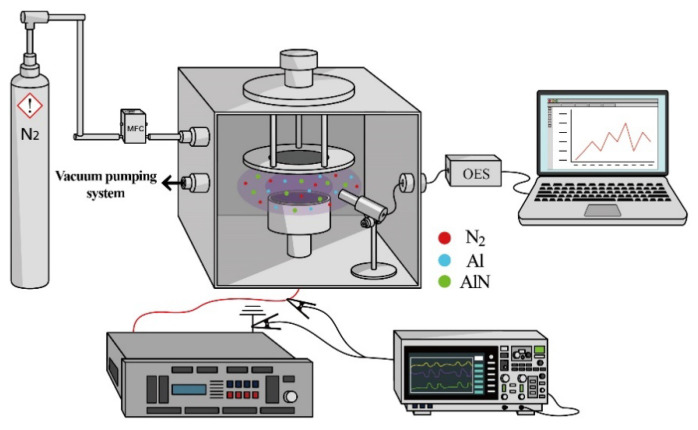
The schematic diagram of the sputter chamber.

Figure 2 shows a high vacuum chamber and equipment used in the experiment. In this study, P-type Si (100) substrates (4-inch) were used to deposit 500–600 nm thick of AlN films in a reactive DC magnetron sputtering system with an asymmetric bipolar-pulsed DC power generator (HPP-1K5A01KAT, Delta Incorporated, Taiwan) under a base pressure of 8 × 10^−6^ torr at room temperature. A pure aluminum target (5N) with 101.6 mm diameter is used and the distance from the target to the substrate is 80 mm. The deposition time is 30 min at various sputtering pulse parameters. The interaction variables with the factor levels and PVD process setup for a total of 15 experiments are listed in Table 1. Moreover, we use optical emission spectroscopy (OES) detector (model: SE2020-025-FUVN, OtO Photonics, Taiwan@ resolution 1.2–6.0 nm FWHM) in the process to record the plasma spectral intensity at wavelength 250–850 nm with a sampling rate of 1/3 Hz and integration time to 50 ms. The OES data played a crucial role in the modeling process. OES measurements provided a real-time, in-situ monitoring of the plasma conditions during the deposition process, enabling us to collect valuable information on the chemical composition, excitation, ionization states, and other plasma characteristics. In addition to the role, OES data serves as a valuable training dataset for the thin film state classification model. By employing the spectral information obtained from OES measurements, the model can identify and distinguish between different thin film states based on their unique spectral signatures. This allows for the development of a robust machine learning algorithm, which can effectively classify thin films based on their properties and deposition conditions.

Each Si substrate was cleaned with acetone, isopropyl alcohol, and deionized water in an ultrasonic oscillator for 3 min to remove grease and particles. Then, substrates were pickled with 2% hydrofluoric acid (HF) for one minute to remove surface native oxide. Additionally, prior to the deposition, Ar+ ion bombardment was applied to clean the Al target surface to remove the oxide layer, contamination layer, and any adsorbates [22].

### 2.2. Measurements

X-ray diffraction (XRD) measurements were performed by a model of D8 Advance (Bruker US). θ/2θ scans were recorded on a monochromator-less diffractometer delivering Cu-Kα (λ = 1.5418 Å) radiation which was operated at 40 kV and 40 mA in 34°–40°. FWHM data were calculated from AlN (002) peak measured from the center of AlN film.

The surface morphologies of AlN films were characterized by an atomic force microscope (AFM, Nanoview 1000, FSM, China) equipped with an n-type contact cantilever silicon tip in radius size of 7 nm. Use the tapping mode to measure the surface roughness of thin films to avoid surface scratches on test pieces and to obtain higher quality images.

**Figure 3:**
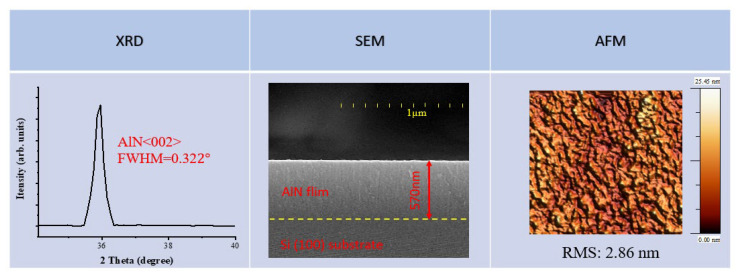
Typical thin film quality measurements by XRD, SEM and AFM for sample S8.

The characterization of film microstructure and thickness was carried out by Field emission scanning electron microscopy (FE-SEM, JSM-7000F, Japan). The cross-section of AlN films was taken from the observed AlN (002) columnar nanocrystalline, confirming its quality [23] and measurements of the film’s thickness at ×20,000 magnification. Furthermore, we take the film surface morphology images at ×80,000 magnification to calculate the grain size. Figure 3 shows the results of the three techniques utilized in detecting the microstructure of the thin films of sample S8.

### 2.3. Box–Behnken Design Coupled with RSM on Experiments

Box–Behnken design coupled with RSM was employed to setup a design of experiment (DOE) for optimizing the quality of AlN films. Box–Behnken method is more efficient and requires fewer experiments to analyze the results [24,25]. In addition, through two and three-dimensional analysis, the influence of pulse parameters on films can be more intuitively found. In this experiment, the independent variables were based on three pulsed DC generator parameters in reverse voltage (X1), pulse frequency (X2) and duty cycle (X3). RSM layouts were obtained from statistical software (Minitab 19, Scientific Formosa, Inc.) that can generate surface plots from experimental data to describe trend under specific pulse parameters to model and analyze problems in which responses are influenced by three input variables.

### 2.4. Dimensionality Reduction and GBDT-Based Algorithm

**Figure 4:**
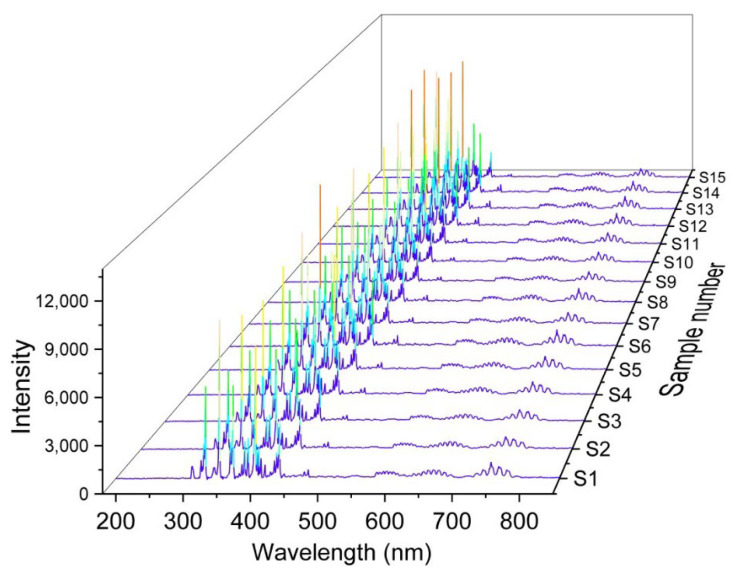
Optical emission spectra that were measured separately were affected by pulse parameters. Spectrum data were integrated with the plasma’s time (0–30 min) in S1–S15.

In this study, we used the OES datasets for machine learning model training; Figure 4 shows 15 sets of OES data affected by pulse parameters with 9966 datasets. Each dataset consists of 1900 features. When the number of dimensions exceeds three, it becomes increasingly difficult to visualize the relationships between variables. To relieve high-dimension issues mentioned, we use principal component analysis (PCA) dimensionality reduction techniques to preprocess the OES datasets. The PCA is a linear transformation that seeks to find the eigenvectors (principal components) and eigenvalues (variances of the principal components) of the covariance matrix which is calculated using the original dataset. The first principal component corresponds to the eigenvector with the largest eigenvalue, and each subsequent principal component corresponds to the eigenvector with the next largest eigenvalue. These principal components are orthogonal to each other. It means that they are uncorrelated. The following is a general equation for PCA in Equation (1). Where variable *N* is the number of all data in the dataset (assuming sample *x*), *v*_1_ and *v*_2_ are two features in sample *x*, and *v*_i1_ and *v*_i2_ represent the *i*-th sample of *v*_1_ and *v*_2_ in the sample *x*, respectively.

(1) $$Cov\;\left(v_{1},v_{2}\right)=\sum_{i=1}^{N}\frac{\left(v_{i1}-v_{1}\right)\left(v_{i2}-v_{2}\right)}{N-1}$$

We used CatBoost machine learning model for training after PCA data preprocessing. CatBoost model is a gradient boosting decision tree-based (GBDT) machine learning algorithm developed by Yandex in 2017. This algorithm model uses an ensemble method for the improvement of the accuracy of the model by combining the predictions of multiple decision trees. It can prevent overfitting by controlling the growth of decision trees and has good generalization ability. Compared to other GBDT models, the CatBoost model has better explainability, allowing direct observation of the impact of each decision tree. It also supports an adaptive loss function, which can adapt to different task requirements, such as binary classification, multi-classification, regression, etc. The equation for the final prediction can be represented by Equation (2). Where *F*(*x*) is the final prediction, *W_j_* is the weight assigned to the *j*-th decision tree, *h_j_*(*x*) is the prediction made by the *j*-th decision tree for the dataset (assuming sample *x*), and the variable *M* is the number of decision tree splits.

(2) $$F\left(x\right)=\sum_{j}^{M}W_{j}\times h_{j}\left(x\right)$$

## 3. Results and Discussion

AlN films were fabricated using a reactive DC magnetron sputtering with a total of 15 different DOEs combined with three DC pulse parameters in reverse voltage (50–90 V), pulse frequency (100–250 kHz), and duty cycle (80–90%). Pulse parameters have a direct effect on the state of the plasma. Therefore, we optimized the power parameters to obtain better quality of AlN films. Table 2 presents the 15-run experimental designs from Box–Behnken method.

### 3.1. Characterization

**Figure 5:**
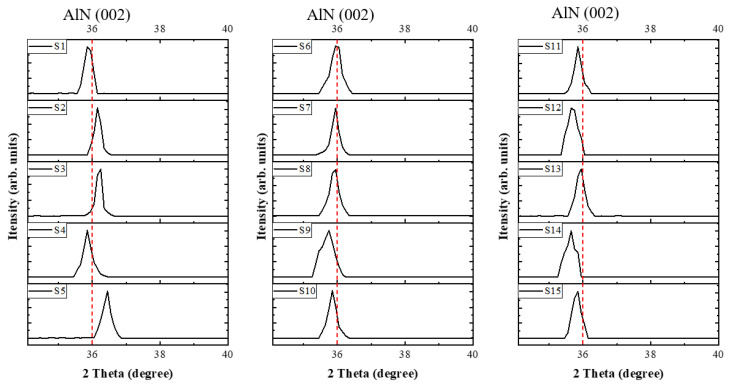
XRD (θ/2θ scan) spectra of AlN films (S1–S15) deposited at various DC pulsed parameters of 50–90 V reverse voltage, 100–250 kHz pulse frequency, 80–90% duty cycle as well as N_2_ concentration of 100% and flow rate of 60 sccm.

Figure 5 shows XRD (θ/2θ scan) spectra of AlN films (S1–S15) deposited at various sputtering pulse parameters. We observed prominent peaks corresponding to the AlN crystallite phases, which are critical in assessing the quality and crystallinity of the films. These peaks include the (100) and (002) planes, commonly reported in the literature for hexagonal wurtzite AlN films. It can provide insights into the film’s overall purity and the effectiveness of the sputtering process. The microstructures of AlN films were analyzed extensively by XRD scanning in the range of 34°–40°. The impact of three DC pulsed parameters on film FWHM was studied by varying the design of pulse parameters. It can be clearly observed that each group has an obvious AlN 002 peak at about 36°. The film FWHM was obtained by calculations from a Lorentz curve fit in the experimental data between 34° and 38°. Among them, FWHM in fifteen sets of experiments is between 0.1°–0.506°. We observed that experiment S3 has the minimum FWHM value, indicating that the AlN film has better crystallinity under this parameter. In an asymmetric bipolar pulsed-DC reactive sputtering, the reverse voltage is critical. It has to maintain an appropriate value without back sputtering and to prevent the target from poisoning due to the arc causing fine dust particles deposition. The asymmetric bipolar pulsed DC is also dependent on pulse frequency. To prevent the build-up potential (voltage) from crossing the parasitic capacitors, the pulse frequency has to maintain enough beyond its breakdown voltage [26,27,28].

**Figure 6:**
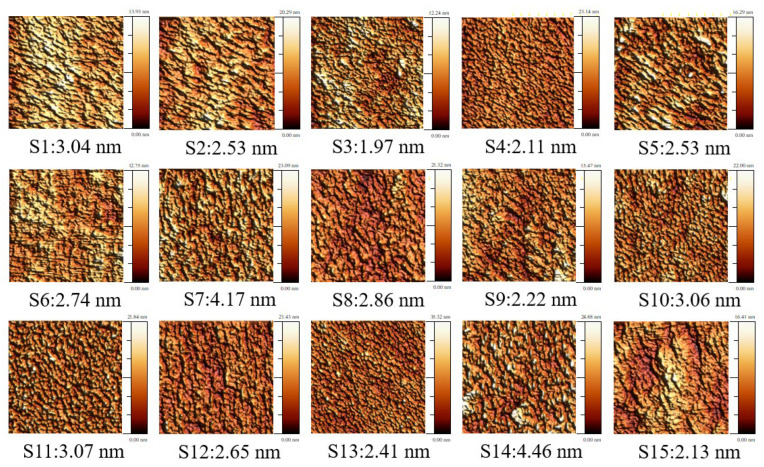
AFM images of AlN films (S1–S15 in RMS surface roughness) as deposited on Si (100) substrates.

**Figure 7:**
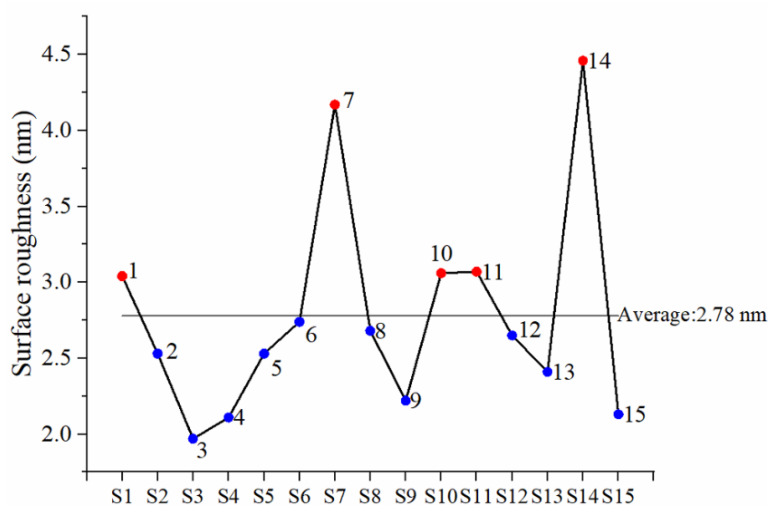
The surface roughness with root-mean-square (RMS) after process completion for the sputtered AlN films in the various pulse parameters as shown in Figure 6 (S1–S15).

AlN is an excellent piezoelectric material for electroacoustic devices for example, bulk acoustic wave (BAW) and surface acoustic wave (SAW) devices. However, uneven surfaces or microstructure, such as cracks and voids, will cause transmission loss. Furthermore, if the roughness of the surface is greater than the traveling wavelength, the acoustic waves cannot pass through the material surface, which will affect the SAW speed [5,29]. Therefore, the surface roughness is extremely important to the device performance; we use AFM (tapping mode) and FE-SEM to measure the surface roughness, the microscopic crystal morphology, and the cross-sectional structure of AlN films. The results are shown in Table 3. The surface roughness data are recorded in the center of the film using the tapping mode and the measurement range is 5 µm × 1.2 µm. This mode will bring the probe closer to the sample and increase the amplitude. The amplitude change is used to obtain high-resolution images when contacting the sample. The advantage is that the contact force between the probe and the sample can be reduced, thereby preventing the film surface from scratching. AFM images of AlN films were shown in Figure 6. The RMS surface roughness values on 15 sets of AlN films were 1.97 nm–4.46 nm. According to Figure 7, experiments S1, S7, S10, S11, and S14, which display roughness above the average, all have a reverse voltage greater than or equal to 70 V in common.

**Figure 8:**
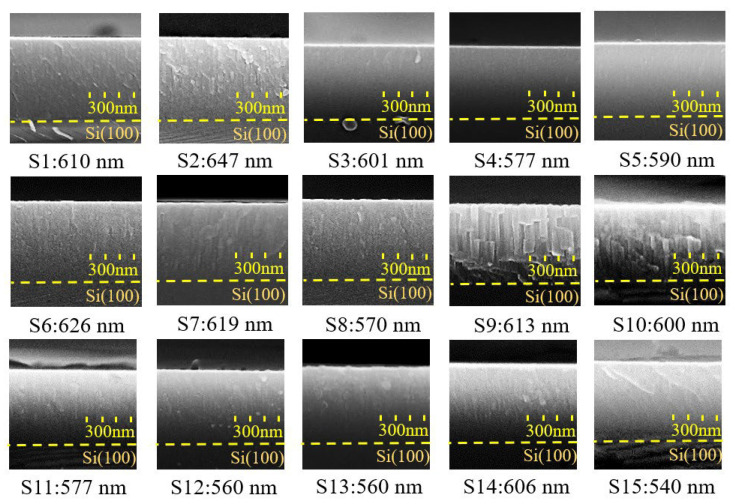
The cross-sectional micrographs of FE-SEM on AlN thin films (S1–S15 in film thickness).

**Figure 9:**
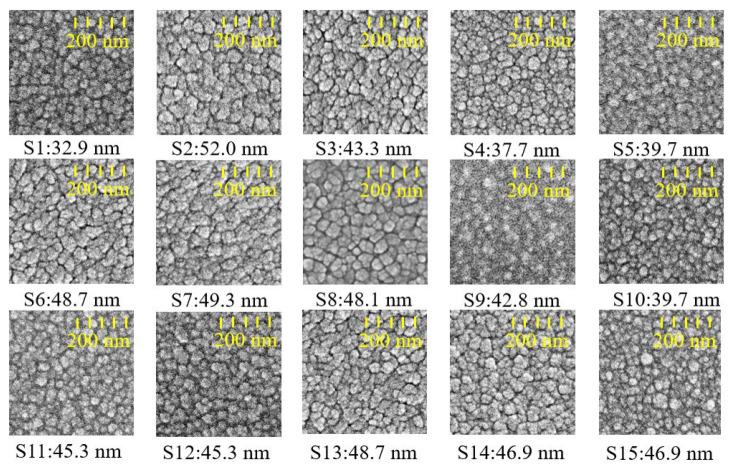
The top-views of SEM images on AlN films (S1–S15 in average grain size) as deposited on Si (100) substrates.

Figure 8 shows the cross-sectional micrographs of AlN films deposited on silicon (100) substrates taken by FE-SEM. The 30 min process exhibited a film thickness between 540–647 nm and a deposition rate of 18.0–21.6 nm/min. It was observed that when the duty cycle was set at a small value (S11, S12, and S15), resulting in a shorter sputtering period (ton), the deposition rate was significantly lower. The duty cycle is proportional to the sputtering period in a complete current waveform. The value represented by the duty cycle is presented in Equation (3). In addition, the increase in pulse frequency will also increase plasma activity. The top-views of AlN films are depicted in Figure 9. Thin film morphology confirms the XRD data discussed above; the (002) oriented films showed pebble-like structures with average grain sizes ranging between 30.9–52.0 nm, indicating lower surface mobility of adatoms under pure nitrogen conditions [30] and a clear columnar structure is also shown in the cross-sectional micrographs in Figure 8.

(3) $$DutyCycle=\frac{\left(t_{on}\right)}{\left(t_{on}\right)+\left(t_{rev}\right)}\times 100\%$$

### 3.2. Statistical Analysis and Optimization

In this experiment, the Box–Behnken factorial outline with three factors was selected to analyze the three pulse parameters. A linear response equation was generated from the measured and calculated FWHM values. Multiple regression analyses were performed on the experimental data and response variables. We established a second-order polynomial equation, as shown in Equation (4), to explain the relationship between pulse parameters and film FWHM. Equation (4) can predict the response of each factor at three levels (−1, 0, +1), the high levels were labeled as +1, and the low levels were labeled as −1. The reverse voltage (*X*_1_) and duty cycle (*X*_3_) have a more significant linear impact. The coefficients are 0.1064 and −0.238, respectively. In the interaction between pulse parameters, the reverse voltage and duty cycle also have the most significant impact, and its value is −0.001302. According to the above results, the duty cycle (*X*_3_) has the greatest influence, followed by reverse voltage (*X*_1_) and pulse frequency (*X*_2_).

(4) $$\begin{array}{ll} Y\left(FWHM\right) & =5.75+0.1064X_{1}+0.01129X_{2}-0.238X_{3}\\ & -0.000014X_{2}*X_{2}+0.00204X_{3}*X_{3}\\ & +0.000029X_{1}*X_{2}-0.001302X_{1}*X_{3}-0.000103X_{2}*X_{3} \end{array}$$

All responses from the results of variance (ANOVA) analysis by a quadratic polynomial model with a corresponding valid model obtained from Minitab 19 software are depicted in Table 4. In statistical hypothesis testing, the significance level is typically set at 0.05 or below, meaning that results with a *p*-value greater than 0.05 are generally not considered statistically significant. In general, the smaller the *p*-value is, the more significant the results are, with a *p*-value of less than 0.05 indicating strong evidence against the null hypothesis [31,32]. It can be seen from the variance analysis of the regression model that the quadratic model has a certain significance, the *p*-value is 0.04, and the R-squared value is 85.91%, suggesting that this model has strong explanatory power. Only 14.09% of the total variation remains unexplained, indicating it is a good fit model between the prediction and the data. In addition, the smaller *p*-value of the squared and interaction terms of the independent variables indicates a more significant impact of these terms in the model. However, even the R-square (adj) value is 67.13%, and a lower value may suggest a simpler model with fewer variables. The model can still provide valuable insights and predictive power for the response variable.

**Figure 10:**
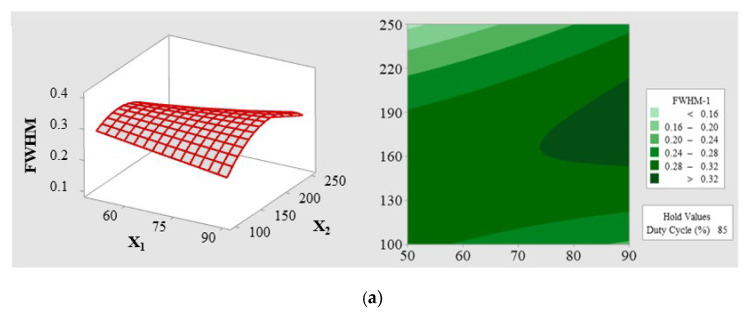

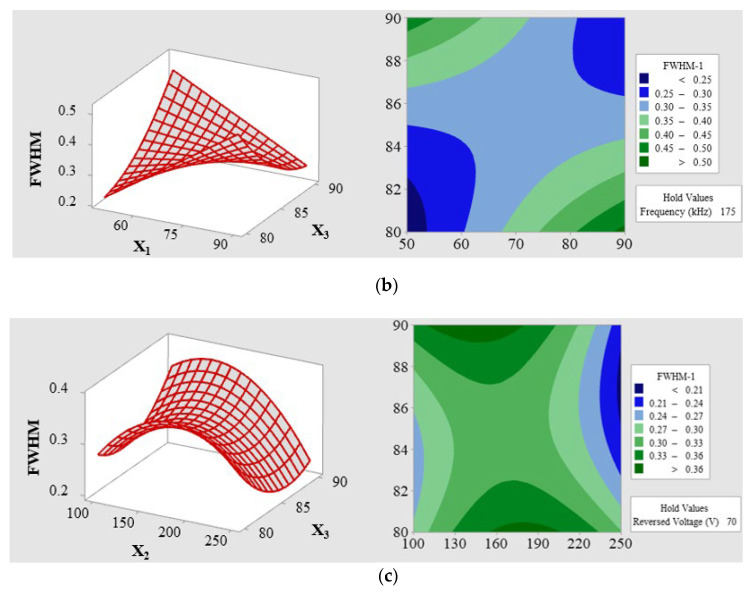
2D views (factor vs. level) and 3D views. (**a**) 3D in X_1_*X_2_: frequency interacting with reverse voltage, X_3_ remains a constant; (**b**) 3D in X_1_*X_3_: duty cycle interacting with reverse voltage, X_2_ remains a constant; and (**c**) 3D in X_2_*X_3_: duty cycle interacting with frequency, X_3_ remains a constant. Here, factors are reverse voltage (X_1_), pulse frequency (X_2_), and duty cycle (X_3_).

Furthermore, to better visualize and evaluate the effects of pulse parameters on optimizing the FWHM, we converted the above-mentioned second-order polynomial equation (Equation (4)) into 2D and 3D response surface plots. These plots provide an intuitive representation of the relationships among the three main variables (reverse voltage, pulse frequency, and duty cycle), shown in Figure 10.

In constructing these plots, one parameter was fixed at a medium level, while the FWHM was analyzed as a function of the other two interaction factors. Specifically, Figure 10a illustrates the response of FWHM when the reverse voltage interacts with the pulse frequency, with the duty cycle held constant at a medium level (85%). The plot shows that better FWHM values are achieved when the reverse voltage is at a low level, and the pulse frequency is at a high level. Similarly, Figure 10b displays the response of the reverse voltage interacting with the duty cycle while the pulse frequency is maintained at a medium level (175 kHz). Lastly, Figure 10c presents the response of the pulse frequency interacting with the duty cycle, with the reverse voltage fixed at a medium level (70 V).

By examining these response surface plots, we can gain a deeper understanding of the complex interactions among the pulse parameters, which aids in optimizing the FWHM based on the second-order polynomial equation (Equation (4)).

In summary, the response of the interaction of multiple variables in the above RSM diagram is integrated, and a set of optimized parameters is obtained, presented in Table 5. The best results obtained in this research are reverse voltage 50 V, pulse frequency 250 kHz, and duty cycle 80.6061%. The estimated FWHM value is 0.11°. Due to the much higher mobility of electrons compared to ions, the positive voltage is usually set at 10–20% of the negative working voltage to achieve complete discharge. However, it must be kept low enough to prevent the occurrence of back sputtering and reduce the risk of arc and target poisoning. Deposition at higher pulse frequencies enhances plasma activity and concentration, resulting in better film uniformity. Additionally, a higher duty cycle could result in an increase in the sputtering period, which may lead to a better deposition rate. However, it could also cause a breakdown voltage exceeding the parasitic capacitors on target, resulting in arcing and subsequently deteriorating the quality of the deposited film [28,33].

### 3.3. Data Preprocessing and Model Training

**Figure 11:**
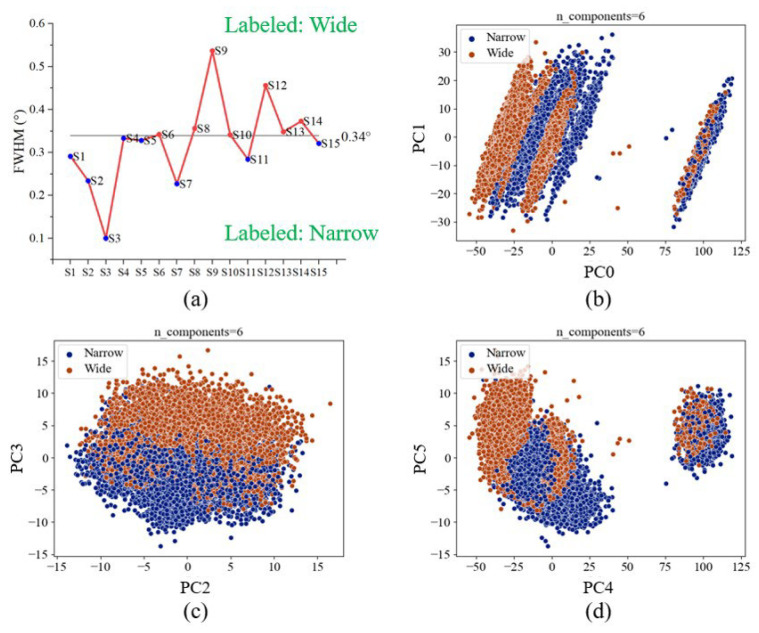
(**a**) The schematics of the labeled data of the FWHM model. (**b**) The first vs. second principal components (PC0 and PC1). (**c**) The third vs. fourth principal components (PC2 and PC3). (**d**) The fifth vs. sixth principal components (PC4 and PC5).

**Figure 12:**
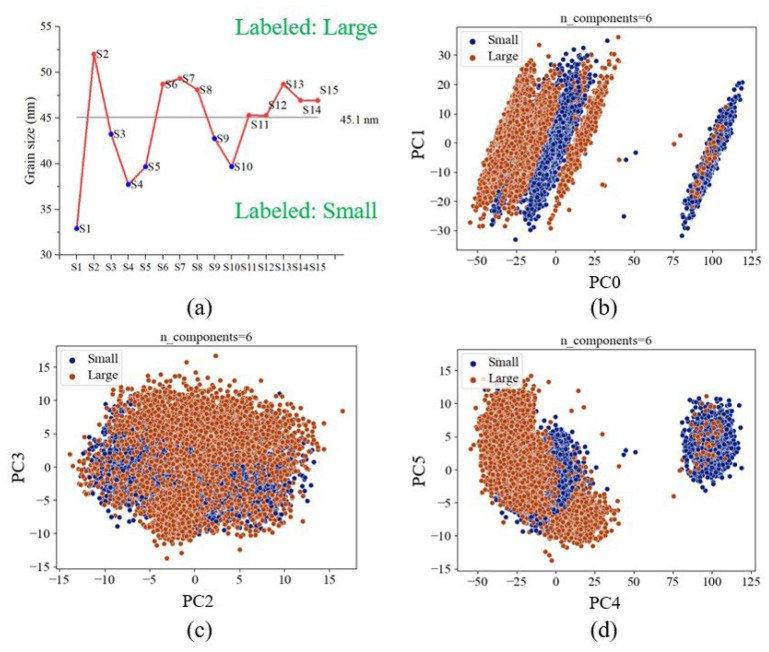
(**a**) The schematic of the labeled data of the grain size model. (**b**) The first vs. second principal components (PC0 and PC1). (**c**) The third vs. fourth principal components (PC2 and PC3). (**d**) The fifth vs. sixth principal components (PC4 and PC5).

We trained two CatBoost models to classify FWHM and grain, respectively. For the FWHM model, if the measured data exceeds the average value of 0.34 degrees, we label it as “Wide”, and the opposite is labeled as “Narrow”. The schematics of the labeled data are shown in Figure 11a. For the grain size model, if the measured data exceed the average value of 45.1 nm, we label it as “Large”, and the opposite is labeled as “Small”. The schematic of the labeled data is shown in Figure 12a.

For data preprocessing, we use PCA to reduce the dimensionality of OES data from 1900 features to 6 features (PC0 to PC5). After PCA dimensionality reduction, the data is transformed into a new set of principal components (PCs) that capture the maximum variance in the original dataset. The cumulative variance is the sum of variances of the principal components (eigenvalue of the covariance matrix illustrated in Section 2.4). The means of the cumulative variances increase as the number of principal components increases. Typically, we hope to select the smallest number of principal components that capture a large percentage of the total variance, to reduce the dimensionality of the data and simultaneously preserve the information as much as possible. The cumulative variances from PC0 to PC5 are 73.59%, 79.12%, 82.51%, 84.73%, 89.12%, and 91.14%, respectively. The cumulative variances mean the total variation in a dataset explained by each principal component. From the calculation results using Python software (scikit-learn toolkit), we can interpret 91.14% of the features in the dataset through six principal components. The dimensionality data can improve the calculating time and eliminate outliers and visualization effectively after dimensionality reduction. From the visualized results, the same labeled data are well clustered. The well-clustered data refer to a dataset where the data points are grouped closely together after transformed space. When the data is well-clustered, it becomes easier for machine learning models to learn the relationships between the features and the target variable. As a result, machine learning models trained on well-clustered data tend to have better training performance, including higher accuracy and faster convergence during training. Figure 11b,c and Figure 12b,c, show the results of dimensionality reduction. The blue and orange dots signify two distinct types of data. Despite the proximity of the clusters, the results of the dimensionality reduction reveal a clear and discernible clustering pattern. This noticeable separation between the data groups suggests that the accuracy of the prediction model is likely to be enhanced. Consequently, the clustering visualized in the figure serves as a valuable indicator of the model’s potential performance.

The machine learning model training process typically involves two important steps: data split and hyperparameter tuning. The data split involves dividing the available dataset into training, validation, and testing sets. The training data are used to train the model, while validation data are used to evaluate the model’s performance during training and select the best hyperparameters. Finally, the test data are used to evaluate the final performance of the trained model after hyperparameter tuning. Hyperparameter tuning involves selecting the best value of hyperparameters that maximizes the model’s performance on validation data. Once the best set of hyperparameters is selected, the model can be trained again using the entire training data, and the final performance can be evaluated on the test data. Following these two steps can ensure that the machine learning model is properly trained and optimized for the given dataset.

For CatBoost model training, the datasets have been split into the training dataset 70%, the validation dataset 15%, and the testing dataset 15%. This distribution to splitting dataset into training, validation, and test subsets allows for a sufficiently large training set (70%) to develop and refine the prediction model while also providing separate validation (15%) and test (15%) sets to assess its performance and generalization capabilities. The validation set is useful for tuning model parameters and preventing overfitting, while the test set serves as a final evaluation to ensure that the model performs well on new or unseen data. This method of dataset partitioning is commonly used in machine learning applications and is considered good practice for robust model evaluation. We mainly tuned the parameters of iteration and learning rate. We fixed the parameters of max_depth = 3 and l2_leaf_reg = 4, and both the FWHM and grain size models are set to be the same iteration (=1000) and learning rate (=0.01). The max_depth and l2_leaf_reg are the hyperparameters in the CatBoost. The parameters of the max_depth can control the maximum depth of each decision tree in the ensemble model (CatBoost in this study). The l2_leaf_reg can control the L2 regularization to the leaf weights of each decision tree in the ensemble. The best value for max_depth and l2_leaf_reg depends on the scale and complexity of the dataset. Then the trained model for FWHM and grain size received the performance metrics as shown in Table 6. The FWHM model had a recall of 89.52%, precision of 94.51%, F1-score of 91.95%, and accuracy of 90.67%. The grain size model had a recall of 86.40%, precision of 79.61%, F1-score of 82.87%, and accuracy of 82.07%. Four basic terms that refer to four possible outcomes of binary classification in machine learning are the values of *TP* (true positive), *TN* (true negative), *FP* (false positive), and *FN* (false negative). TP and TN are correct prediction results, and *FP* and *FN* are incorrect. The values of *TP*, *FP*, *FN*, and *TN* as accuracy, precision, recall, and F1-score, respectively, can be adopted to evaluate the performance of a prediction model. Details are illustrated in Equations (5)–(7).

(5) $$Recall=\frac{TP}{TP+FN}$$

(6) $$Precision=\frac{TP}{TP+FP}$$

(7) $$F1\text{-}score=\frac{2\times Recall\times Precision}{Recall+Precision}$$

## 4. Conclusions

We use Box–Behnken experimental design to reduce the number of experiments and collected data to develop a reliable model, which enables us to make more appropriate adjustments to parameters according to device requirements, thus saving a lot of time and effort in improving its quality. We obtained the optimized conditions of the pulse parameters of the best FWHM as reverse voltage: 50 V, pulse frequency: 250 kHz, and duty cycle: 80.6061%. In addition, using AFM and SEM to analyze the film’s microstructure, the relationship among surface roughness, deposition rate and pulse parameters was obtained. We compared relevant literature and found that the FWHM ranged from 0.28°–0.36° and decreased with a decrease in duty cycle (20–70%). However, the pulse frequency used in the literature was 100 kHz, which may not be sufficient to achieve the desired plasma activity and concentration [34]. Compared with other studies using asymmetric bipolar pulse deposition of AlN thin films, our results also showed better performance [35]. This study also successfully trained two models that can predict film FWHM and grain size. Machine learning algorithms can analyze amounts of data and identify OES data features that are difficult for humans to find. Accurately predicting a semiconductor deposition process can reduce the number of failed runs and improve overall efficiency. It can make more accurate predictions about the deposition process, reducing the time and cost required for testing. Moreover, machine learning algorithms can provide insights into the deposition process that may not be apparent through traditional analysis methods. This can help engineers make more informed decisions about the process and improve the overall quality of the semiconductor products produced. In the future, we will develop more expertise in plasma physics, data analysis, and machine learning, as well as access to the necessary equipment, and data (parameters) to develop a complete plasma information-based virtual metrology (PI-VM) system. This can provide real-time feedback to engineers, enabling them to optimize the semiconductor processing and improve yields. We know that uncertainty factors can impact the prediction model, specifically the blank signals and noise measured by the OES during the setup period. Suppose these noise components are inadvertently included in the model training process without proper attention or filtering. In that case, the model’s prediction accuracy may be compromised, resulting in errors and reduced reliability of the predictions. The novelty of this study is that it verifies the application of the PCA method to rapidly reduce the dimensionality of OES data and validates the feasibility of using reduced OES data for predicting film quality (FWHM and grain size). Since the prediction model belongs to a classification model that classifies two classes (above or below the average value), it cannot be used to predict the actual value of FWHM and grain size. A predictive model of actual values is planned for the future.

## 5. Future Work

One of the primary goals for our future studies is to investigate the density of AlN films, as it is a crucial parameter for semiconductor applications and is directly related to the sputtering parameters. We will explore opportunities for collaboration with other laboratories or institutions to access this technology. By utilizing X-ray reflectivity (XRR) equipment, and measurements, we can estimate the density of the AlN films and examine its relationship with the sputtering conditions. Furthermore, we will seek to refine our prediction model by incorporating additional parameters, such as film stress, adhesion, and thermal conductivity, to achieve a more comprehensive understanding of the film properties. Additionally, we plan to compare the results obtained using our optimized parameters with previously published studies to validate the effectiveness of our approach. Through these efforts, we aim to enhance the optimization of sputtering parameters for AlN film deposition in semiconductor applications and contribute to the broader body of knowledge in this field.

In the upcoming experiments, we will model and simulate the problem of target erosion. First, develop a computer model to simulate the sputtering process and predict the effect of target erosion on film properties. The model incorporates parameters such as target material, deposition conditions, and erosion rate Incorporate parameters such as target material, deposition conditions, and erosion rate. Then, use the model to simulate the sputtering process under varying degrees of target erosion and predict the corresponding film properties. The software expected to be used for computer modeling and simulation of sputtering processes is COMSOL Multiphysics.

## Figures and Tables

**Table 1:** The interaction variables of the factor levels and sputtering conditions by experimental design.

Manipulated Variables	Units	Signs	Levels		
			Low (−1)	Medium (0)	High (+1)
Reverse Voltage	V	X_1_	50	70	90
Pulse Frequency	kHz	X_2_	100	175	250
Duty Cycle	%	X_3_	80	85	90
**Response variable**					
FWHM	°	Y			
**Sputtering parameter**	**Units**	**Values**			
Pressure	mTorr	2			
Power	W	1200			
Deposition time	min	30			
Distance	cm	8			
Substrate temperature	°C	R.T.			
Total gas flow rate	sccm	60			
N_2_	sccm	60			

**Table 2:** 15-run experimental designs from Box–Behnken method.

Run	Factors		
	X_1_	X_2_	X_3_
S1	0	−1	+1
S2	+1	+1	0
S3	−1	+1	0
S4	−1	−1	0
S5	+1	−1	0
S6	0	0	0
S7	0	+1	+1
S8	0	0	0
S9	−1	0	+1
S10	+1	0	+1
S11	0	−1	−1
S12	+1	0	−1
S13	0	0	0
S14	0	+1	−1
S15	−1	0	−1

**Table 3:** Results of characterization for 15 experimental sets.

Sample	002 Peak FWHM (°)	Surface Roughness (nm)	Deposition Rate (nm/min)	Grain Size (nm)
S1	0.29	3.04	20.33	32.9
S2	0.23	2.53	21.57	52.0
S3	0.10	1.97	20.03	43.3
S4	0.31	2.11	19.23	37.7
S5	0.27	2.53	19.67	39.7
S6	0.34	2.74	20.87	48.7
S7	0.23	4.17	20.63	49.3
S8	0.32	2.68	19.00	48.1
S9	0.51	2.22	20.43	42.8
S10	0.27	3.06	20.00	39.7
S11	0.28	3.07	19.23	45.3
S12	0.47	2.65	18.67	45.3
S13	0.32	2.76	18.67	48.7
S14	0.38	4.46	20.20	46.9
S15	0.19	2.13	18.00	46.9

**Table 4:** A summary from response surface regression analysis.

Summary of Model					
R-squared	R-squared (adj)		R-squared (pred)		
85.91%	67.13%		N.D.		
ANOVA					
**Source**	**DF ^a^**	**Adj SS ^b^**	**Adj MS ^c^**	**F-Value**	***p*-Value**
* Model Linear X_1_ X_2_ X_3_ * Square X_2_X_2_ X_3_X_3_ * 2-Way Interaction X_1_X_2_ X_1_X_3_ X_2_X_3_ Error Lack of fit Pure Error Total	8 3 1 1 1 2 1 1 3 1 1 1 6 4 2 14	0.124731 0.007574 0.002016 0.005512 0.000045 0.035624 0.023623 0.009661 0.081533 0.007744 0.067860 0.005929 0.020452 0.020168 0.000285 0.145183	0.015591 0.002525 0.002016 0.005515 0.000045 0.017812 0.023623 0.009661 0.027178 0.007744 0.067860 0.005929 0.003409 0.005042 0.000142	4.57 0.74 0.59 1.62 0.01 5.23 6.93 2.83 7.97 2.27 19.91 1.74 35.42	0.040 0.565 0.471 0.251 0.912 0.049 0.039 0.143 0.016 0.182 0.004 0.235 0.028

Footnotes for ^a^ DF: degree of freedom; ^b^ Adj SS: adjusted sum of squares; ^c^ Adj MS: adjusted mean square; * the importance of correlation parameters.

**Table 5:** Multiple response prediction.

Factor X_1_ Reverse Voltage	Factor X_2_ Pulse Frequency	Factor X_3_ Duty Cycle	Response FWHM
50 V	250 kHz	80.6061%	0.110969°

**Table 6:** Basic terms and critical indicators of two trained models.

Goal	Fundamental Terms				Performance Metrics			
	TP [%]	FP [%]	FN [%]	TN [%]	Recall [%]	Precision [%]	F1-Score [%]	Accuracy [%]
FWHM	53.32	3.09	6.23	37.35	89.52	94.51	91.95	90.67
Grain Size	43.36	11.10	6.82	38.70	86.40	79.61.51	82.87	82.07

## Data Availability

The data presented in this study are available in article.
